# A Novel Piezoresistive Accelerometer with SPBs to Improve the Tradeoff between the Sensitivity and the Resonant Frequency

**DOI:** 10.3390/s16020210

**Published:** 2016-02-06

**Authors:** Yu Xu, Libo Zhao, Zhuangde Jiang, Jianjun Ding, Niancai Peng, Yulong Zhao

**Affiliations:** State Key Laboratory for Manufacturing Systems Engineering, Collaborative Innovation Center of Suzhou Nano Science and Technology, Xi’an Jiaotong University, Xi’an 710049, China; cactusxy@stu.xjtu.edu.cn (Y.X.); zdjiang@mail.xjtu.edu.cn (Z.J.); dingjianjun@126.com (J.D.); ncpeng@mail.xjtu.edu.cn (N.P.); zhaoyulong@mail.xjtu.edu.cn (Y.Z.)

**Keywords:** piezoresistive accelerometer, sensitivity, resonant frequency, SPBs

## Abstract

For improving the tradeoff between the sensitivity and the resonant frequency of piezoresistive accelerometers, the dependency between the stress of the piezoresistor and the displacement of the structure is taken into consideration in this paper. In order to weaken the dependency, a novel structure with suspended piezoresistive beams (SPBs) is designed, and a theoretical model is established for calculating the location of SPBs, the stress of SPBs and the resonant frequency of the whole structure. Finite element method (FEM) simulations, comparative simulations and experiments are carried out to verify the good agreement with the theoretical model. It is demonstrated that increasing the sensitivity greatly without sacrificing the resonant frequency is possible in the piezoresistive accelerometer design. Therefore, the proposed structure with SPBs is potentially a novel option for improving the tradeoff between the sensitivity and the resonant frequency of piezoresistive accelerometers.

## 1. Introduction

Vibration signals usually present different parameters, including velocity, displacement or acceleration, that can be measured by a velocity sensor, a displacement probe or an accelerometer, respectively. In practical applications, accelerometers is some of the most popular vibration sensors because of their convenience. When designing an accelerometer, the sensitivity and resonant frequency are often chosen as the most significant performance characteristics to optimize the structure. Due to the working principle based on the spring-mass system [1,2,3], the maximum displacement of the system is often regarded as an important manifestation of the accelerometer sensitivity. For example, piezoresistive or piezoelectric accelerometers count on the electrical variations caused by a stress, but the outputs in essence arise from the displacement of the spring-mass system [4,5,6,7,8,9,10,11,12,13]. In capacitive accelerometers, the output is directly affected by the displacement of the spring-mass system [14,15,16]. Therefore, there is a dependency between the displacement of the spring-mass system and the sensitivity of an accelerometer. The tradeoff between the displacement and resonant frequency of the spring-mass system is transformed into that between the sensitivity and resonant frequency of the accelerometer due to this dependency.

For improving the tradeoff between the resonant frequency and sensitivity in the accelerometer design, many studies have focused on new sensing mechanisms such as silicon nanowires in order to obtain higher sensitivity [17,18,19,20]. However, these methods depend on new processes or new materials, and they are difficult to manufacture in mass production. Therefore, many researchers are still concentrated on the geometry design to optimize accelerometers [21,22,23,24,25,26,27,28,29].

In general, the product of the square of resonant frequency and the sensitivity is often chosen as a figure of merit (FOM) to evaluate the accelerometer performance [30]. The FOM can be expressed by the following equation:

(1) $$\mathrm{FOM}=S\omega^{2}$$

where *S* is the sensitivity of the accelerometer, *ω* is the circular resonant frequency of the accelerometer. However, it is difficult to simultaneously increase the sensitivity and resonant frequency of an accelerometer.

In theory, the accelerometer detects the applied acceleration by using a proof mass based on the Newton’s second law. Therefore, most accelerometers are designed as a second-order mass-spring-damper system, for which the following equation can be formulated:

(2) $$ma_{applied}=m\overset{..}{x_{o}}+c\dot{x_{o}}+kx_{o}$$

where *m* is the mass of the proof mass, *k* is the spring constant, *c* is the damping ratio, *a_applied_* is the applied acceleration, and *x*_o_ is the displacement of the proof mass.

In the static response, the displacement of the proof mass is equivalent to the force generated by acceleration of the mass to the spring constant, an equation that can be expressed as:

(3) $$x_{o}=\frac{ma_{applied}}{k}=\frac{a_{applied}}{\omega^{2}}$$

For the piezoresistive accelerometer, the sensitivity relies on the stress distribution of piezoresistors instead of the displacement of the accelerometer. Assuming a full Wheatstone bridge, the sensitivity of a piezoresistive accelerometer can be given by:

(4) $$S=\frac{U_{out}}{a_{applied}}\frac{1}{U_{applied}}$$

where *U_out_* is the output voltage and *U_applied_* is the applied voltage to the Wheatstone bridge. According to the working principle of the piezoresistive accelerometer, the output voltage is given by:

(5) $$U_{out}=\pi \sigma U_{applied}$$

where *π* is the piezoresistive coefficient of the piezoresistors, *σ* presents the stress of the piezoresistors. They are both affected by the material property. From Equations (4) and (5), we can have:

(6) $$S=\frac{\pi \sigma }{a_{applied}}$$

Therefore, Equations (1), (3) and (6) can be combined to:

(7) $$\text{FOM}=S\frac{a_{applied}}{x_{o}}=\pi \frac{\sigma }{x_{o}}$$

**Figure 1:**
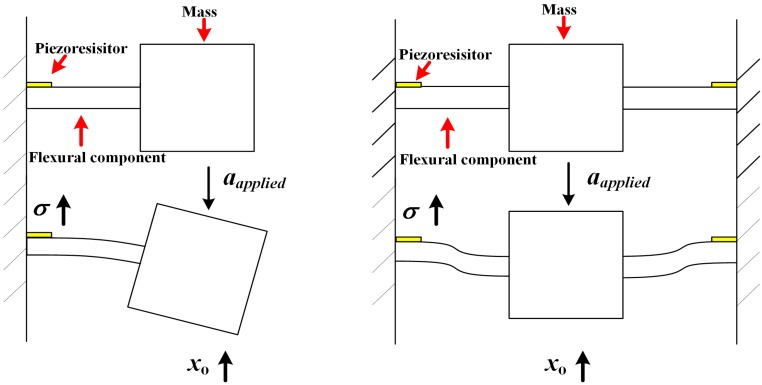
The dependency between *x*_o_ and σ in the conventional piezoresistive accelerometer.

Instead of increasing the sensitivity or resonant frequency individually in Equation (1), it is more reasonable to obtain a better stress per displacement ratio (denoted as *σ*/*x*_o_) to improve FOM in Equation (7). However, in the conventional piezoresistive accelerometer shown in Figure 1, the piezoresistors are often embedded into a flexural component of the accelerometer such as a beam. The stress distribution of the piezoresistors usually depends on the displacement of the accelerometer under an applied acceleration, which indirectly results in a coupling relationship between *x*_o_ and *S*. Meanwhile, the dependency mentioned above can be also considered as the dependency between *x*_o_ and *σ*. Therefore, it is difficult to weaken the dependency in conventional piezoresistive accelerometer designs.

In order to improve the tradeoff between the sensitivity and the resonant frequency, a novel structure with SPBs for the purpose of weakening the dependency between *x*_o_ and *σ* is proposed in this paper to optimize the performance of piezoresistive accelerometers. Firstly, a theoretical model based on the Euler-Bernoulli beam theory is derived. The theoretical formulas are also investigated to calculate the location of SPBs, the stress in SPBs and the resonant frequency of the structure. Secondly, the theoretical results are verified by finite element method (FEM) simulations. Meanwhile, the experimental results are also discussed to compare with the theoretical results and FEM results. Finally, the proposed structure is compared to that with same dimensions but without SPBs for further discussion.

## 2. Theory

### 2.1. Theoretical Structure

**Figure 2:**
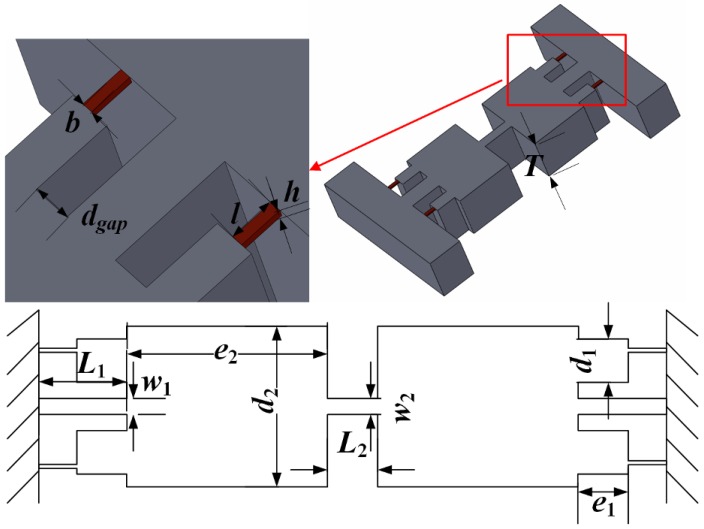
The schematic of the proposed structure.

As shown in Figure 2, the schematic of the proposed structure consists with two masses, four SPBs (*b* *×* *l* *×* *h*) as the piezoresistors, two supporting beams (*w*_1_ × *L*_1_ × *T*) and one hinge (*w*_2_ *×* *L*_2_ *×* *T*). Each mass contains two balance masses (*e*_1_ *×* *d*_1_ *×* *T*) and one proof mass (*e*_2_ *×* *d*_2_ *×* *T*) which are considered as a rigid body. The SPB dimensions and *d*_gap_ are determined by the fabrication capability. Other parameters are determined by the SPB dimensions and measurement requirements including the measurement sensitivity and resonant frequency. Four SPBs are symmetrically located on two sides of the supporting beams to form the Wheatstone bridge for sensing acceleration.

**Figure 3:**
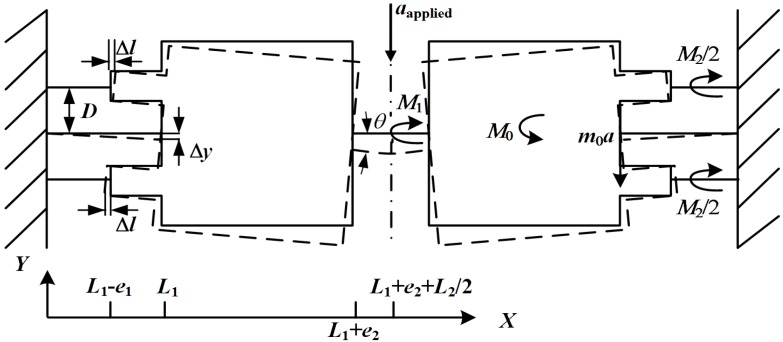
Simplified structure of the proposed structure.

Figure 3 shows the simplified structure of the proposed structure. When an acceleration is applied to the structure in the in-plane direction, both masses move upward and downward in parallel, and this results in a displacement of the supporting beams (denoted as Δ*y*) and the rotations of the mass (denoted as *θ*). The stress distributions of the piezoresistors in the proposed structure should be a pure axial-deformation in order to avoid the stress losses to improve the sensitivity. The condition to achieve the pure axial-deformation of the SPBs depends on the distance (denoted as *D*) in *y*-axis from the center plane of the SPBs to that of the supporting beam. If the structure is properly designed, the *y*-direction displacement at the end of the SPBs can be offset by the rotation of the mass. Finally, only a pure axial-deformation (denoted as Δ*l*) is generated in each SPB.

Due to the axial symmetry of the proposed structure, only half of the structure is taken into considered in the analytical structure. Based on the Euler-Bernoulli beam theory and small deflection principle, when the acceleration is loaded to the structure in *y*-axis, the differential equations of force moment balance are expressed as:

(8) $$EI_{1}Y_{1}^{"}(x)=m_{0}(L_{1}-x)a+M_{0}-M_{1}-M_{2}\;(0<x<L_{1})$$

(9) $$EI_{2}Y_{2}^{"}(x)=M_{1}\;(L_{1}+e_{2}<x<L_{1}+e_{2}+L_{2}/2)$$

The boundary conditions are:

(10) $$Y_{1}(0)=0$$

(11) $$Y_{1}^{\prime }(0)=0$$

(12) $$Y_{1}^{\prime }(L_{1})=Y_{2}^{"}(L_{1}+e_{2})$$

(13) $$Y_{2}^{\prime }(L_{1}+e_{2}+L_{2}/2)=0$$

where *E* is Young’s modulus of silicon. *ρ* is the density of silicon. *I*_1_ = *w*_1_^3^*T*/12 and *I*_2_ = *w*_2_^3^*T*/12 are the cross sectional moment of the supporting beam and the hinge, respectively. *Y*_1_(*x*) and *Y*_2_(*x*) are the displacements of the supporting beam and the hinge. *m*_0_ is the total mass of one proof mass and two balance masses. *M*_0_, *M*_1_ and *M*_2_ are the total inertial moment of the mass, the bending moment of the hinge and the restrictive moment of the SPBs, respectively.

The terms *m*_0_, *M*_0_ and *M*_1_ can be expressed as:

(14) $$m_{0}=(2\rho e_{1}d_{1}T+\rho e_{2}d_{2}T)$$

(15) $$M_{0}=(\frac{1}{2}\rho e_{2}^{2}d_{2}T-\rho e_{1}^{2}d_{1}T)a$$

(16) $$M_{1}=\frac{I_{2}}{I_{1}L_{2}/2-I_{2}L_{1}}(\frac{1}{2}m_{0}L_{1}^{2}a+M_{0}L_{1}-M_{2}L_{1})$$

When the SPBs achieve the pure axial-deformation, *M*_2_ can be expressed as:

(17) $$M_{2}=2FD=2k_{\text{sensing}}\Delta lD=2k_{\text{sensing}}D^{2}\theta$$

where Δ*l* = *Dθ* is the pure axial-deformation of the SPBs. *F* = *k*_sensing_ Δ*l* is the axial force of the SPB and *k*_sensing_ = *Ebh*/*l* is the spring constant of the SPB.

### 2.2. The Location of the SPBs for Achieving Axial-Deformation

Based on the theoretical structure mentioned above, *θ* can be calculated as:

(18) $$\theta =\frac{a}{\frac{EI_{1}}{L_{1}}+\frac{EI_{2}}{L_{2}/2}+\frac{2Ebh}{l}D^{2}}(\frac{1}{2}m_{0}L_{1}+\frac{1}{2}\rho e_{2}^{2}d_{2}T-\rho e_{1}^{2}d_{1}T)$$

When the SPBs have achieved axial-deformation, the *y*-axis displacements of the SPBs ends connected with the balance mass should be zero. When *θ* << 1 (*θ* ≈ tan*θ*), the *y*-axis displacement can be expressed as:

(19) $$\Delta y_{\text{SPB}}=Y_{1}(L_{1})-e_{1}\theta =\Delta y-e_{1}\theta =0$$

Therefore, *θ* can be also expressed as:

(20) $$\theta =\frac{a}{\frac{EI_{1}e_{1}}{L_{1}^{2}}+\frac{EI_{2}}{L_{2}}+\frac{Ebh}{l}D^{2}}(\frac{1}{3}m_{0}L_{1}+\frac{1}{4}\rho e_{2}^{2}d_{2}T-\frac{1}{2}\rho e_{1}^{2}d_{1}T)$$

From Equation (18) to Equation (20), the location of the SPBs can be expressed as:

(21) $$\begin{array}{l} D=\sqrt{\frac{l}{bh}\frac{1}{m_{0}}\frac{6}{L_{1}}}\times [(\frac{I_{1}e_{1}}{L_{1}^{2}}+\frac{I_{2}}{L_{2}})(\frac{1}{2}m_{0}L_{1}+\frac{1}{2}\rho e_{2}^{2}d_{2}T-\rho e_{1}^{2}d_{1}T)-\\ (\frac{I_{1}}{L_{1}}+\frac{I_{2}}{L_{2}/2})(\frac{1}{3}m_{0}L_{1}+\frac{1}{4}\rho e_{2}^{2}d_{2}T-\frac{1}{2}\rho e_{1}^{2}d_{1}T){]}^{\frac{1}{2}} \end{array}$$

As seen in Equation (21), it is clear that the location of the SPBs is only affected by the geometrical parameters and material density.

### 2.3. The Stress of the SPBs

After the location of the SPBs and *θ* are determined, it is easy to know the stress σ of the SPBs as:

(22) $$\sigma =E\frac{\Delta l}{l}=E\frac{D\theta }{l}$$

Compared with other piezoresistive accelerometers, the stress of the SPBs depends not only on *θ*, which is directly proportional to the displacement of the structure (or, inversely proportion to the resonant frequency of the structure), but also the location of the SPBs.

### 2.4. The Frequency of the Structure

Based on the Rayleigh-Ritz method, the resonant frequency of the structure is calculated as:

(23) $$f=\frac{1}{2\pi }\sqrt{\frac{\int_{0}^{L_{1}}EI_{1}{\left[Y_{1}^{"}(x)\right]}^{2}dx+\int_{L_{1}+e_{2}}^{L_{1}+e_{2}+L_{2}/2}EI_{2}{\left[Y_{2}^{"}(x)\right]}^{2}+2k_{\text{sensing}}\Delta l^{2}}{\int_{L_{1}}^{L_{1}+e_{2}}\rho d_{2}t{\left[Y_{1}(L_{1})+Y_{1}^{\prime }(L_{1})\times (x-L_{1})\right]}^{2}dx+2\int_{L_{1}-e_{1}}^{L_{1}}\rho d_{1}t{\left[Y_{1}(L_{1})+Y_{1}^{\prime }(L_{1})\times (x-L_{1})\right]}^{2}dx}}$$

In Equation (23), the numerator consists of the elastic energies of the supporting beam, half the hinge and two SPBs. The denominator includes the kinetic energies of the proof mass and the two balance masses. From Δ*l* = *Dθ*, it is clear that the resonant frequency of the structure is also influenced by the location of the SPBs.

## 3. Theoretical Validation

In this section, the validity and the accuracy of theoretical structure for calculating *D* with various geometrical parameters are discussed. Meanwhile, the effects of *D* on σ and *f* by geometry modification are presented. The simulations are carried out and compared with theoretical results by the commercial FEM software (ANSYS15.0, ANSYS Inc., Canonsburg, PA, USA). In all simulations, the applied acceleration and the material properties are kept with the same values. The applied acceleration is 100 g and the material properties include the density of 2330 kg/m^3^, the elastic modulus of 170 GPa and the Poisson’s ratio of *v* = 0.28. The values of *T, b*, *h*, *l* and *d*_gap_ are assumed to be constant. The initial geometrical dimensions are shown in Table 1.

**Table 1:** The initial geometrical dimensions.

	*w*_1_	*L*_1_	*e*_1_	*d*_1_	*e*_2_	*d*_2_	*w*_2_	*L*_2_	*T*	*b*	*h*	*l*	*d_gap_*
Initial dimensions (μm)	70	500	430	395	1600	1000	30	500	310	5	10	70	70

### 3.1. The Effects of Various Geometrical Parameters on D

**Figure 4:**
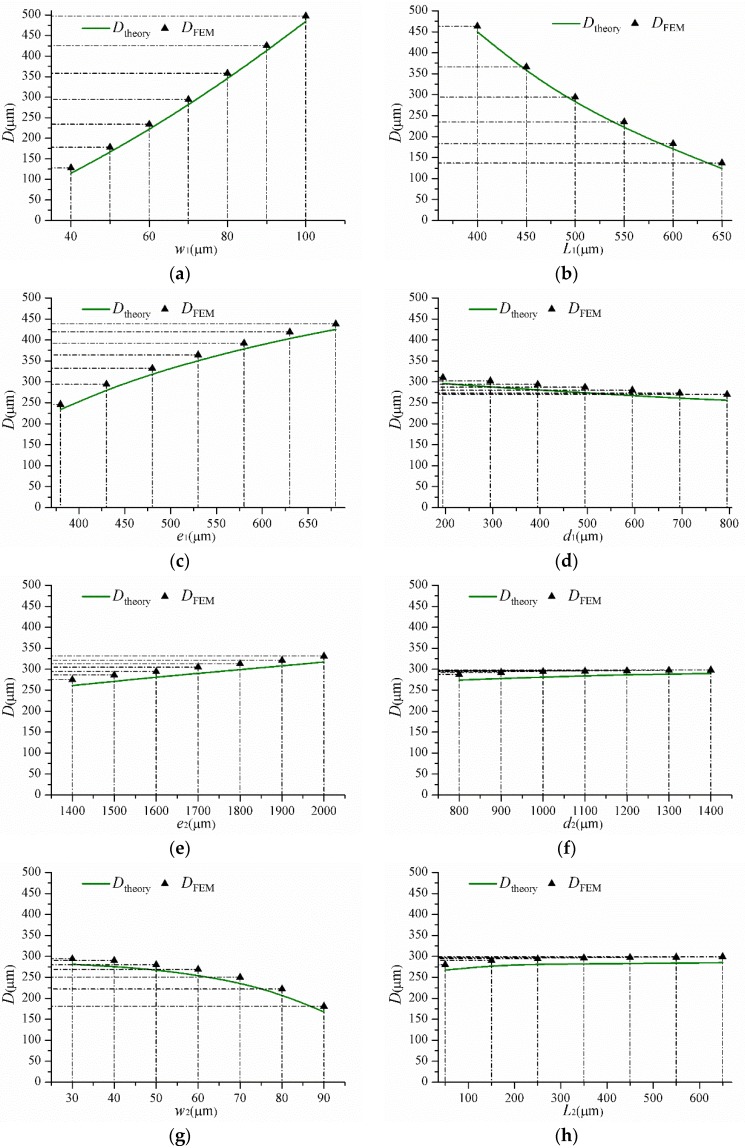
The effects of different geometrical parameters on *D.* (**a**) The effect of *w*_1_; (**b**) The effect of *L*_1_; (**c**) The effect of *e*_1_; (**d**) The effect of *d*_1_; (**e**) The effect of *e*_2_; (**f**) The effect of *d*_2_; (**g**) The effect of *w*_2_; (**h**) The effect of *L*_2_.

In this section, the accuracy of Equation (21) for calculating the locations of the SPBs with various geometrical dimensions is discussed. Figure 4 explains the influences of different geometrical parameters on *D*. The results demonstrate clearly that Equation (21) is remarkably precise for calculating *D*. The geometrical dimension ranges and maximum relative errors between the simulation and theoretical results are shown in Table 2.

**Table 2:** The geometrical dimension ranges and maximum relative errors.

	***w*_1_**	***L*_1_**	***e*_1_**	***d*_1_**
Geometrical dismension range (μm)	40–100	400–650	380–680	195–795
Max relative error	10.9%	9.4%	4.8%	5.1%
	***e*_2_**	***d*_2_**	***w*_2_**	***L*_2_**
Geometrical dismension range (μm)	1400–2000	800–1400	30–90	50–650
Max relative error	5.0%	4.5%	7.1%	4.6%

As shown in Figure 4a,b, it is clear that the value of *D* increases with an increasing *w*_1_ or a decreasing *L*_1_. In other word, the value of *D* increases with the enhancement of the supporting beam stiffness. It can be seen from Figure 4c,d that the value of *D* increases with an increasing *e*_1_ or a decreasing *d*_1_. Although it is difficult to summarize the effect of the balance mass, it is shown from the results that the balance mass still plays an important role in controlling *D*.

Figure 4e,f demonstrate the value of *D* increases with an increasing *e*_2_ or an increasing *d*_2_. Namely, *D* increases with the increasing weight of the proof mass. From Figure 4g,h, we can see that the value of *D* increases with a decreasing *w*_2_ or an increasing *L*_2_. In contrast to the supporting beam, *D* increases with decreasing the hinge stiffness in Figure 4g,h.

### 3.2. The Effects of Various Geometrical Parameters on σ and f

**Figure 5:**
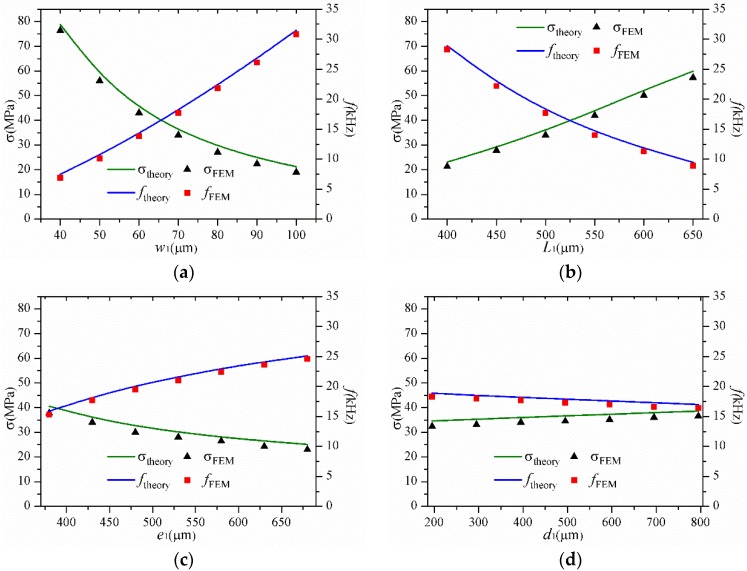

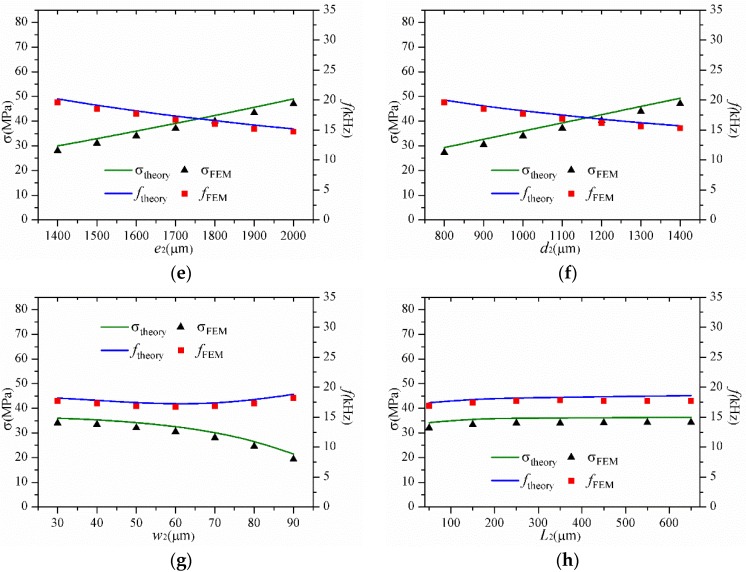
The effects of different geometrical parameters on *σ* and *f.* (**a**) The effect of *w*_1_; (**b**) The effect of *L*_1_; (**c**) The effect of *e*_1_; (**d**) The effect of *d*_1_; (**e**) The effect of *e*_2_; (**f**) The effect of *d*_2_; (**g**) The effect of *w*_2_; (**h**) The effect of *L*_2_.

The influences of different geometrical parameters on σ and *f* are displayed in Figure 5. It is clearly demonstrated that Equations (22) and (23) are remarkably precise for calculating σ and *f*. The geometrical dimension ranges are the same as those in Table 2. The maximum relative errors between the simulation and theoretical results are shown in Table 3.

**Table 3:** The maximum relative errors.

	*w*_1_	*L*_1_	*e*_1_	*d*_1_	*e*_2_	*d*_2_	*w*_2_	*L*_2_
Maximum relative error of σ	10.3%	7.1%	8.3%	6.3%	6.7%	6.8%	9.4%	6.4%
Max relative error of *f*	8.0%	6.2%	3.7%	3.5%	3.2%	2.6%	2.9%	2.8%

The results indicate that the increasing stiffness of the supporting beam results in a decreasing *σ* or an increasing *f* in Figure 5a,b. In Figure 5c,d, a decreasing *e*_1_ or an increasing *d*_1_ led to an increasing *σ* and a decreasing *f*. The increasing weight of the proof mass results in an increasing *σ* and a decreasing *f* in Figure 5e,f. However, the results mentioned above show that the dependency between *σ* and *f* still exists. As shown in Figure 5g,h, with the dimensions of the hinge changed, the resonant frequency of the whole structure is affected a little but *σ* is still affected greatly when the dimensions of the hinge are changed. Comparing with other components, it shows a very different relationship between *σ* and *f*. It is easy to know that the increasing *w*_2_ or decreasing *L*_2_ results in the increasing stiffness of the hinge. Meanwhile, the increasing stiffness of the hinge leads to the decreasing *D* as shown in Figure 4g,h. The decreasing *D* makes the SPBs set less mechanical restrictions to the whole structure. The stiffening effect of the SPBs is decreased. Therefore, the stiffening effect between the hinge and the SPBs offsets each other. As a result, the resonant frequency of the whole structure is stabilized by the stiffening effect combined with both the hinge and the SPBs.

From Equation (22), it is easy to know *σ* is affected by *D* and *θ*. *θ* is inversely proportional to the resonant frequency of the structure. It is clearly that *σ* is affected greatly by *D* when the resonant frequency of the whole structure is stabilized. In addition, *σ* is influenced rather more obviously by *w*_2_ than *L*_2_ because of the more effect of *w*_2_ on *D* as shown in Figure 4g,h. In conclusion, the results show that the influences of the SPBs and the hinge play an important role in weakening the tradeoff between *σ* and *f*.

## 4. Results

**Figure 6:**
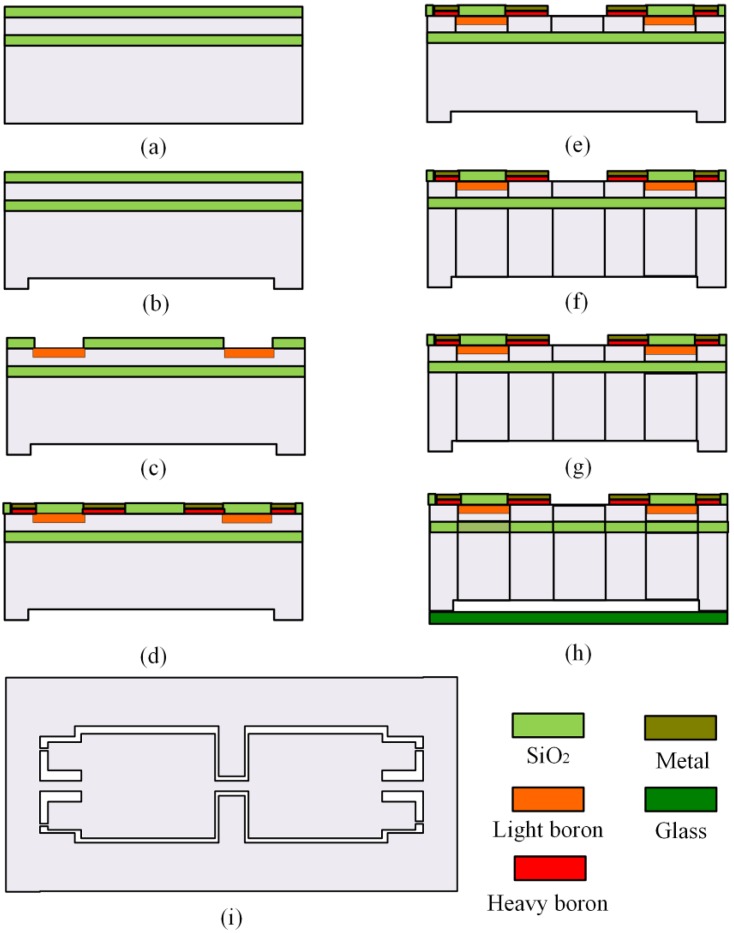
The fabrication process steps of the accelerometer. (**a**) Thermal oxidization; (**b**) ICP for the gap; (**c**) Light boron diffused for piezoresistors; (**d**) Heavy boron diffusion and metallization process; (**e**) RIE for front–side shape; (**f**) DRIE for back–side shape; (**g**) The BOX layer removed; (**h**) Anodic bonding between the SOI wafer and a Pyrex glass slide; (**i**) The top view of the chip.

**Figure 7:**
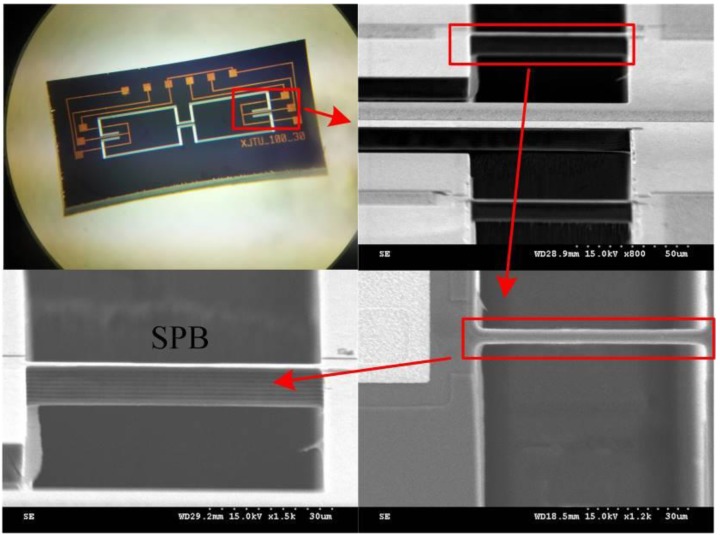
The micrograph of the fabricated chip.

In order to verify the results mentioned above, the proposed structure with SPBs is fabricated with a silicon-on-insulator (SOI) wafer using the micromachining fabrication process. The accelerometer chips are fabricated with three different *w*_2_ values, including *w*_2_ = 30 μm, *w*_2_ = 70 μm and *w*_2_ = 90 μm, respectively. Other dimensions are shown in Table 1. The main fabrication steps of the accelerometer are shown in Figure 6. The fabrication starts with the growth of silicon dioxide in the device layer. In next step, a 4 μm gap is etched at the back side of the wafer using inductively coupled plasma (ICP) technique to ensure the structure move freely after the bottom side is bonded to the Pyrex glass. Then, with the retained SiO_2_ layer as a mask, the light boron is diffused to fabricate the piezoresistors. The nominal sheet resistance after drive-in technology approximates to 220 Ω/ϒ. After the light boron diffusion, the heavy boron diffusion is processed for ohmic contact. A 500 nm-thick Au layer is deposited onto the top surface of the wafer to complete metal electrodes after the contact holes are opened. The reactive ion etching (RIE) technology is processed from the front side to the BOX layer for shaping the SPBs. Then deep RIE (DRIE) is performed from the back side to the BOX layer for shaping the whole structure. Some details about RIE and DRIE are shown in Table 4. The BOX layer is wet etched to release the whole structure by buffered HF. At last, the Pyrex glass to the bottom of the fabricated SOI wafer is packaged by the anodic bonding. Figure 7 shows the micrographs of the fabricated accelerometer chip.

**Table 4:** The details about RIE and DRIE process.

	Gas Flow (sccm)	RF Power (W)	Working Pressure (Pa)	Etching Rate (μm/min)
RIE	SF6:20	20	8	0.4
DRIE	SF_6_:130 O_2_:13 C_4_F_8_:85	Source power:1000 Bias power:20	5.7	4

**Figure 8:**
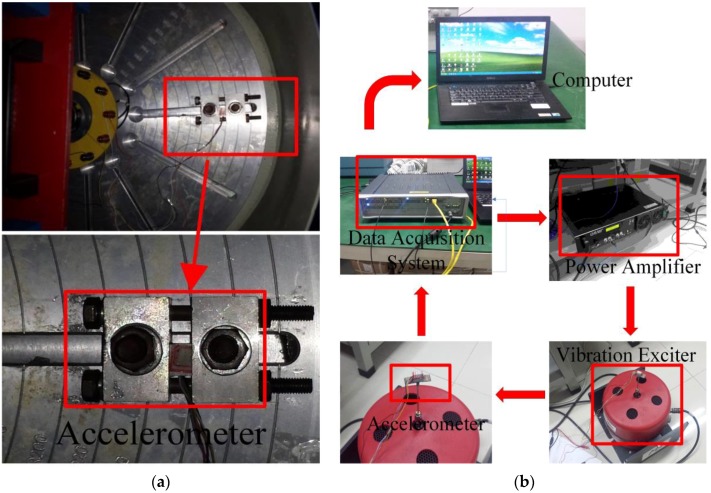
The experiment setups (**a**) The static experiment (**b**) The dynamic experiment.

The fabricated accelerometer chips are tested by static and dynamic experiments, respectively. The experimental setups are shown in Figure 8. First, a stable acceleration centrifugal machine (SY30-3, DONGLING, Suzhou, China) is used in static experiments for measuring the sensitivities of the different accelerometers.

**Figure 9:**
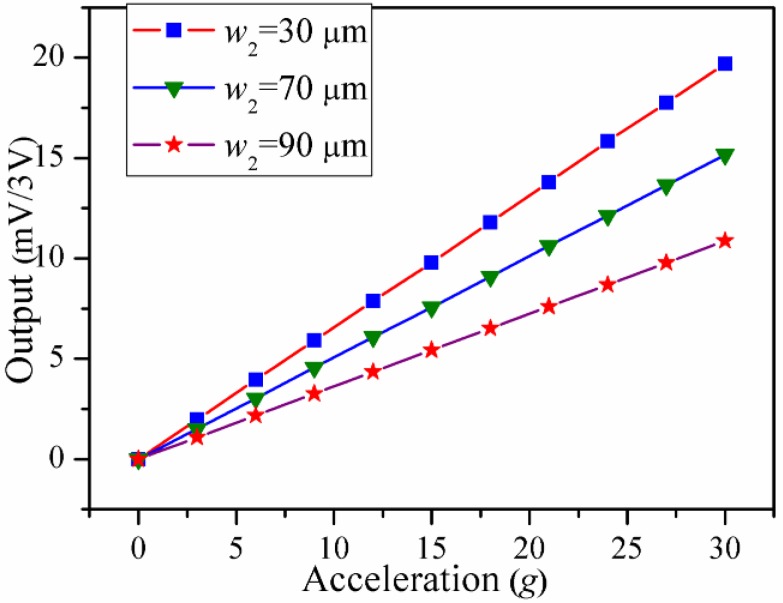
The output voltages *versus* the accelerations.

**Figure 10:**
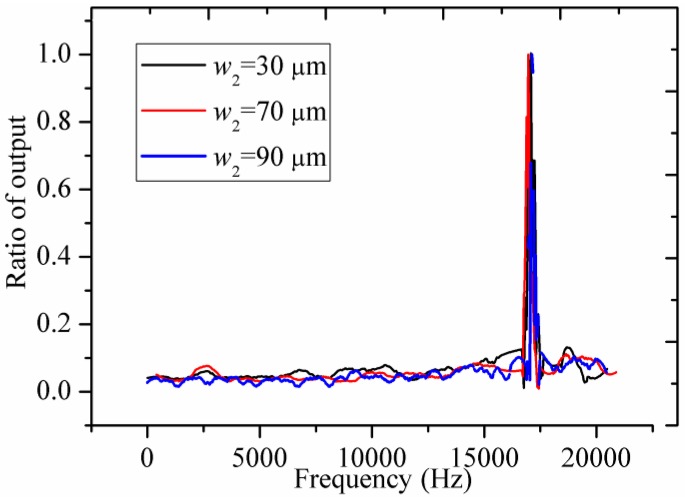
The dynamic curves of output voltage ratios *versus* different frequencies.

The full Wheatstone bridge integrated in each accelerometer is powered with DC 3 V. For safety reasons, the measured range in the static experiments is from 0 to 30 g. Figure 9 shows the output voltages *versus* the accelerations, and then the sensitivities are calculated. Next, the dynamic experiments are investigated by a set of calibration system including a data acquisition system (LMS SCADAS305, Siemens, Munich, Germany), a power amplifier (MB500VI, MB Dynamics, Cleveland, OH, USA), and a vibration exciter (MODAL 110, MB Dynamics, Cleveland, OH, USA). When the swept-frequency of the sinusoidal signal is close to the resonant frequencies of different accelerometers, then the peaks of the dynamic response curves are related to the resonant frequencies. The dynamic curves of the output voltage ratios *versus* different frequencies are shown in Figure 10. The measuring sensitivities and the resonant frequencies are obtained in the Table 5. Based on the experimental results, these accelerometers have different sensitivities, but nearly the same resonant frequencies. The results show a good agreement with the theoretic and FEM results. Therefore, the proposed novel structure is verified as an excellent solution to weaken the tradeoff between the sensitivity and resonant frequency.

**Table 5:** The measuring sensitivities and the resonant frequencies.

	*w*_2_ = 30 μm	*w*_2_ = 70 μm	*w*_2_ = 90 μm
Measuring sensitivity (mV/g/3V)	0.666	0.505	0.363
Resonant frequency (kHz)	17.1	16.9	17.2

## 5. Discussion

**Figure 11:**
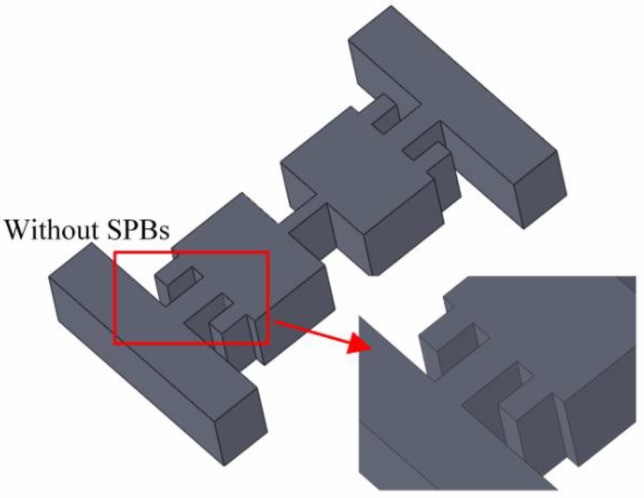
The structure without SPBs.

**Figure 12:**
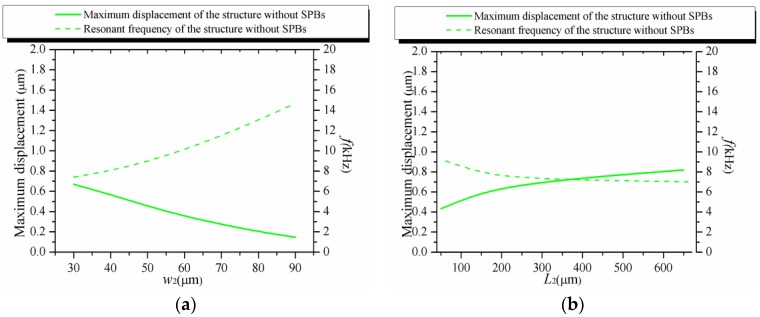
The maximum displacement and the resonant frequency of the structure without the SPBs. (**a**) The effect of *w*_2_; (**b**) The effect of *L*_2_.

**Figure 13:**
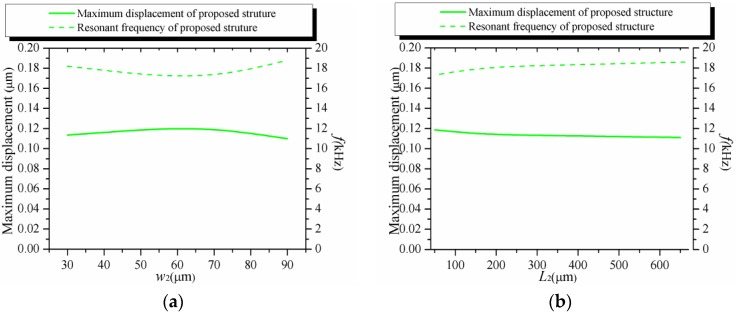
The maximum displacement and the resonant frequency of our proposed structure. (**a**) The effect of *w*_2_; (**b**) The effect of *L*_2_.

In order to further obtain the influences of the combined stiffening effect on stabilizing the resonant frequency, a similar structure without SPBs is shown in Figure 11. In general, the maximum displacement of the structure is inversely proportion to the resonant frequency of the structure. Therefore, the maximum displacement and the resonant frequency of the structure are examined in FEM simulations. The initial dimensions are given in Table 1. The comparisons of the maximum displacement and the resonant frequency of the proposed structure without the SPBs are illustrated in Figure 12, respectively. Like most conventional piezoresistive accelerometer structures, the maximum displacement decreases with the increasing resonant frequency. However, as shown in Figure 13, the displacement and resonant frequency of the proposed structure with SPBs, show little change in a certain range with the variation of *w*_2_ or *L*_2_.

Considering the results illustrated in Figure 12 and Figure 13, the combined stiffening effect between the SPBs and the hinge plays an important role in stabilizing the maximum displacement and the resonant frequency. Combined with the results mentioned above, it is demonstrated that the dependence between the displacement and the sensitivity of accelerometer is weakened by our proposed structure.

## 6. Conclusions

For improving the tradeoff between the sensitivity and the resonant frequency, a novel structure with SPBs is proposed for weakening the dependency between the displacement and *σ* in accelerometer design. FEM simulations, comparative simulations and experiments are carried out to verify the theoretical model, with good agreement. The theoretical and FEM results show that the stress of the piezoresistor increases without sacrificing the resonant frequency when *w*_2_ and *L*_2_ are changed. The experimental results show that the accelerometers fabricated with different *w*_2_ values have significant differences in the sensitivities from 0.363 mV/g/3 V to 0.666 mV/g/3 V but differences of the resonant frequencies from 16.9 kHz to 17.2 kHz are not as evident. It is shown that the tradeoff between the sensitivity and the resonant frequency is changed by the stiffening effect combined with the hinge and the SPBs. In order to further discuss the combined stiffening effect, a similar structure without SPBs is compared with the proposed structure. The comparisons demonstrate that the combined stiffening effect between the SPBs and the hinge plays an indispensable role in weakening the dependence mentioned above. Above all, the proposed structure with SPBs is potentially a better approach for improving the tradeoff between the sensitivity and the resonant frequency to guarantee the high performance of piezoresistive accelerometers.
